# NK Cell-Dependent Antibody-Mediated Immunotherapy Is Improved In Vitro and In Vivo When Combined with Agonists for Toll-like Receptor 2 in Head and Neck Cancer Models

**DOI:** 10.3390/ijms222011057

**Published:** 2021-10-14

**Authors:** Mandy Gruijs, Sonja H. Ganzevles, Marijke Stigter-van Walsum, Richard van der Mast, Monique M. van Ostaijen-ten Dam, Cornelis W. Tuk, Marco W. Schilham, C. René Leemans, Ruud H. Brakenhoff, Marjolein van Egmond, Rieneke van de Ven, Jantine E. Bakema

**Affiliations:** 1 Amsterdam UMC, Department of Molecular Cell Biology and Immunology, Cancer Center Amsterdam—Amsterdam Institute for Infection and Immunity, Vrije Universiteit Amsterdam, De Boelelaan 1117, 1081 HV Amsterdam, The Netherlands; m.gruijs@amsterdamumc.nl (M.G.); r.vandermast@amsterdamumc.nl (R.v.d.M.); cw.tuk@amsterdamumc.nl (C.W.T.); m.vanegmond@amsterdamumc.nl (M.v.E.); 2 Amsterdam UMC, Department of Otolaryngology-Head and Neck Surgery, Cancer Center Amsterdam—Amsterdam Institute for Infection and Immunity, Vrije Universiteit Amsterdam, De Boelelaan 1117, 1081 HV Amsterdam, The Netherlands; s.ganzevles@amsterdamumc.nl (S.H.G.); m.stigter-vanwalsum@amsterdamumc.nl (M.S.-v.W.); cr.leemans@amsterdamumc.nl (C.R.L.); rh.brakenhoff@amsterdamumc.nl (R.H.B.); jab@genmab.com (J.E.B.); 3 Leiden University Medical Center, Department of Pediatrics, Albinusdreef 2, 2333 ZA Leiden, The Netherlands; M.M.van_Ostaijen-ten_Dam@lumc.nl (M.M.v.O.-t.D.); M.W.Schilham@lumc.nl (M.W.S.); 4 Amsterdam UMC, Department of Surgery, Cancer Center Amsterdam, Vrije Universiteit Amsterdam, De Boelelaan 1117, 1081 HV Amsterdam, The Netherlands

**Keywords:** head and neck cancer, immunotherapy, cetuximab, TLR agonists, NK cells, antibody-dependent cellular cytotoxicity, immunosuppression

## Abstract

The immunosuppressive character of head and neck cancers may explain the relatively low response rates to antibody therapy targeting a tumor antigen, such as cetuximab, and anti-PD-1 checkpoint inhibition. Immunostimulatory agents that overcome tumor-derived inhibitory signals could augment therapeutic efficacy, thereby enhancing tumor elimination and improving patient survival. Here, we demonstrate that cetuximab treatment combined with immunostimulatory agonists for Toll-like receptor (TLR) 2 induces profound immune responses. Natural killer (NK) cells, isolated from healthy individuals or patients with head and neck cancer, harbored enhanced cytotoxic capacity and increased tumor-killing potential in vitro. Additionally, combination treatment increased the release of several pro-inflammatory cytokines and chemokines by NK cells. Tumor-bearing mice that received cetuximab and the TLR2 ligand Pam3CSK4 showed increased infiltration of immune cells into the tumors compared to mice that received cetuximab monotherapy, resulting in a significant delay in tumor growth or even complete tumor regression. Moreover, combination treatment resulted in improved overall survival in vivo. In conclusion, combining tumor-targeting antibody-based immunotherapy with TLR stimulation represents a promising treatment strategy to improve the clinical outcomes of cancer patients. This treatment could well be applied together with other therapeutic strategies such as anti-PD-(L)1 checkpoint inhibition to further overcome immunosuppression.

## 1. Introduction

Head and neck squamous cell carcinoma (HNSCC) is an aggressive type of cancer that arises in the mucosal linings covering the upper aerodigestive tract, including the oral cavity, oropharynx, hypopharynx and larynx. Annually, approximately 745,000 patients are diagnosed with HNSCC, and the mortality is over 364,000 patients worldwide [1]. The tumor microenvironment (TME) in HNSCC comprises stromal cells, including endothelial cells and cancer-associated fibroblasts (CAFs). Both tumor cells themselves and CAFs secrete a wide variety of factors that induce immune cell recruitment to the TME. However, the majority of tumor-infiltrating cells have an immunosuppressive phenotype, such as regulatory T-lymphocytes, myeloid-derived suppressor cells (MDSCs) and tumor-associated macrophages (TAMs) [2,3,4,5]. The presence of these cells is associated with poor prognosis [4]. Additionally, tumor-infiltrating cytotoxic T-lymphocytes (CTLs) and natural killer (NK) cells show impaired cytotoxic capacity. Moreover, the TME in HNSCC is characterized by poor antigen presentation, as well as the presence of immunosuppressive factors, including transforming growth factor (TGF) β, interleukin (IL) 6 and IL-10 [5]. Thus, although HNSCC is generally extensively infiltrated by immune cells, it has been shown that the TME can be highly immunosuppressive. Moreover, the immune composition within the TME varies based on the sub-location where the tumor arises or the etiology, i.e., human papilloma virus (HPV)-driven or not [6,7].

In the last decade, immunotherapy has emerged as a treatment option for patients with HNSCC. This was initiated with the FDA approval of cetuximab, a monoclonal antibody (mAb) targeting the epidermal growth factor receptor (EGFR) [8]. Despite overexpression of EGFR on the majority of HNSCC [9], the clinical efficacy of cetuximab treatment is limited in HNSCC patients [8,10,11,12,13]. Currently, treatment with cetuximab, either alone or in combination with conventional radiotherapy or chemotherapy, provides a clinical benefit in 10–20% of patients. Recent attempts to de-escalate the treatment regimen for patients with low-risk HPV+ HNSCC by treating with cetuximab and radiotherapy rather than cisplatin-based chemotherapy and radiotherapy did not favor cetuximab due to a reduction in tumor control [14,15]. Recently, antibodies targeting programmed death receptor 1 (PD-1) and programmed death ligand 1 (PD-L1) have been introduced. Although these antibodies are effective in only 15–20% of HNSCC patients, they have been registered for use in recurrent metastatic disease [16,17,18]. Taken together, it remains important to further enhance the efficacy of immunotherapies. One of the potential therapeutic strategies could be based on improving the efficacy of the immune-mediated mechanisms of cetuximab. Cetuximab blocks the ligand-binding site of EGFR, preventing signaling and subsequent tumor growth [19]. Additionally, cetuximab has the potential to bind IgG Fc receptors (FcγRs), which are expressed on various immune cells, including NK cells. The association of cetuximab with both an EGFR-expressing HNSCC cell and an FcγR-expressing immune cell provides a bridge, inducing antibody-dependent cellular cytotoxicity (ADCC) and subsequent tumor cell elimination [20,21,22,23]. Furthermore, cetuximab activates dendritic cells (DCs), which in turn prime CTLs and, as such, aid in anti-tumor immune responses [24].

The immunosuppressive TME that impairs the cytotoxic capacity of tumor-infiltrating NK cells may contribute to the modest clinical efficacy of cetuximab [25]. Therefore, the combination of cetuximab with immunostimulatory agents and/or immune checkpoint inhibitors may represent a promising strategy to improve the clinical efficacy of cetuximab treatment in HNSCC patients, especially for those patients who are unfit to receive chemotherapy. As clinical trials that study the combination of cetuximab and anti-PD-(L)1 checkpoint inhibition are ongoing, we focused on the addition of immunostimulatory agents to improve cetuximab treatment. Because of their natural immunostimulatory capacity, Toll-like receptors (TLRs) were explored as potential targets in cancer treatment to enhance anti-tumor immune responses [26,27,28,29]. TLRs are pattern recognition receptors (PRRs) that, in the context of cancer, are able to recognize damage-associated molecular patterns (DAMPs). Stimulation of TLRs induces both cytokine secretion and DC maturation, thereby enhancing immune responses [26]. Interestingly, we demonstrated cross-talk between antibody-binding Fc receptors and TLRs on a variety of immune cells, inducing synergistic immune responses that were not observed when either Fc receptors or TLRs were stimulated individually [27,30]. Consequently, in this study, we aimed to improve the activation of NK cells by combining cetuximab with TLR-stimulating agents to enhance their anti-tumor effect.

## 2. Results

### 2.1. Cetuximab Induces Tumor Cell Killing by NK Cells

**Figure 1:**
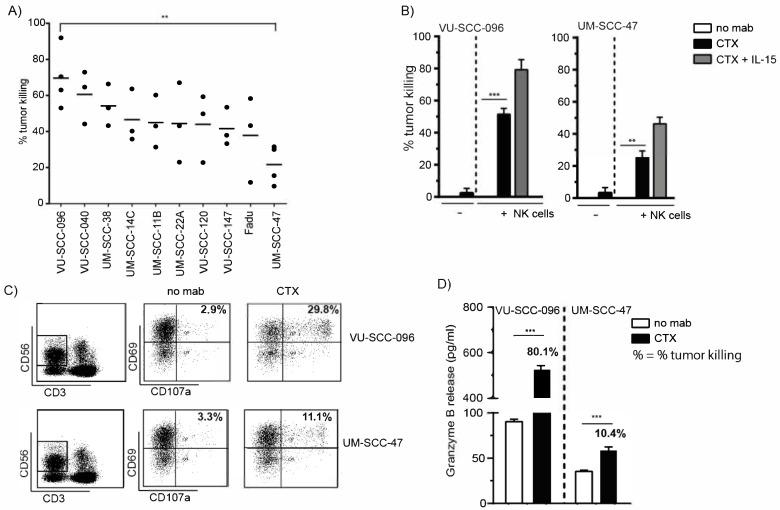
Cetuximab induces tumor cell killing by NK cells. ADCC experiments with different HNSCC cell lines were performed with (**A**) PBMCs (E:T ratio 60:1, 24 h incubation) or (**B**) NK cells (E:T ratio 5:1, 4 h incubation) in the absence or presence of cetuximab (CTX, 0.5 µg/mL) and IL-15. (**A**) Percentage (%) tumor killing in the presence of the anti-EGFR antibody cetuximab (CTX) was calculated compared to untreated conditions, where no cetuximab was added (no mab) (set at 0%). The mean tumor killing for all donor PBMCs is indicated (—) per HNSCC cell line; *n* ≥ 3; ** *p* < 0.01. (**C**) PBMCs were harvested from ADCC experiments described in (**A**). PBMCs were analyzed for % CD69 (activation marker) and CD107a (degranulation marker) double positive NK cells (gated as CD3-CD56+ cells) (upper right square). (**D**) Granzyme B release was measured in supernatants of ADCC experiments described in (**A**) with a sandwich ELISA assay. Corresponding % tumor killing for this particular ADCC experiment is indicated. (**B**–**D**) A minimum of three independent experiments were performed using isolated PBMC from different healthy donors, of which one representative example is shown; bars represent mean ± SD; ** *p* < 0.01; *** *p* < 0.001.

To investigate the cetuximab-mediated elimination of HNSCC cells, ADCC experiments were performed with ten different HNSCC cell lines as target cells and healthy donor peripheral blood mononuclear cells (PBMCs) as effector cells (Figure 1A). Significant differences in the cetuximab-mediated tumor cell killing of HNSCC cell lines by PBMCs were observed, with VU-SCC-096 and UM-SCC-47 being the most and least sensitive HNSCC cell lines, respectively. The difference in the cetuximab-mediated tumor cell killing of VU-SCC-096 cells and UM-SCC-47 cells was also observed in ADCC experiments with purified NK cells (Figure 1B, black bars). The combination of cetuximab with IL-15, an NK cell-activating cytokine, improved tumor cell killing by NK cells (Figure 1B, grey bars). However, the difference in cetuximab-mediated tumor cell killing between the cell lines remained.

The differences in tumor cell killing of the different HNSCC cell lines were not due to donor variation, as all HNSCC cell lines were simultaneously tested with effector cells of the same donor. Moreover, neither the level of EGFR expression on the HNSCC cell lines (Figure S1A and [31,32]) nor the used concentration of cetuximab (Figure S1B) explained the differences in tumor cell killing. In addition, the expression levels of the checkpoint inhibitors PD-L1 and PD-L2 on the tumor cell lines did not explain the differences in tumor cell killing (Figure S1C,D). Therefore, we investigated whether the differences in killing of the different HNSCC cell lines was due to the cytotoxic activity of the NK cells present within the PBMC fraction, measured by the expression of CD69 (activation marker) and CD107a (degranulation marker) on NK cells (Figure 1C). Incubation of VU-SCC-096 cells with NK cells in the presence of cetuximab showed approximately 30% CD69+CD107a+ NK cell levels compared to a rate of less than 12% of CD69+CD107a+ NK cells in the case of UM-SCC-47 cells. This difference was confirmed by increased NK cell cytotoxicity, measured by the release of granzyme B, a tumoricidal and apoptosis-inducing factor [33], in the supernatant of the ADCC experiments (Figure 1D). The elimination of VU-SCC-096 cells in the presence of cetuximab (80% tumor cell killing) induced a tenfold enhanced release of granzyme B compared to UM-SCC-47 cells (10% tumor cell killing). These findings suggest that the tumor cells are capable of modulation of NK cell cytotoxicity.

### 2.2. Co-Activation of FcγRs and TLR2 Enhances Tumor Cell Killing by NK Cells

**Figure 2:**
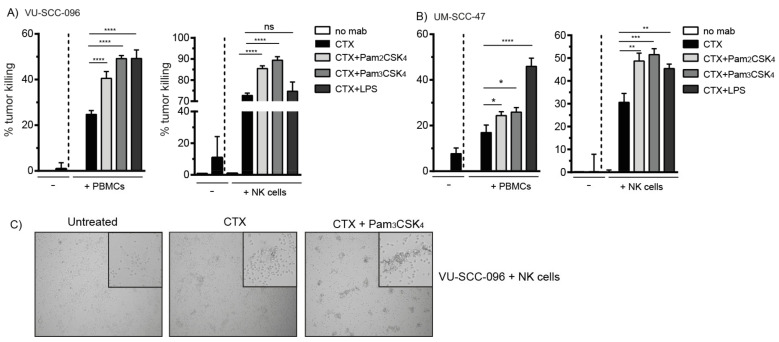
Co-activation of FcγRs and TLR2 enhances tumor cell killing by NK cells. ADCC experiments with the HNSCC cell lines (**A**) VU-SCC-096 (ADCC-sensitive) and (**B**) UM-SCC-47 (ADCC-insensitive) were performed with PBMCs (E:T ratio 60:1, 24 h incubation) or NK cells (E:T ratio 5:1, 4 h incubation). (**A**,**B**) Cetuximab (CTX, 0.5 µg/mL) was added in the absence or presence of agonists for TLR2/6 (Pam2CSK4, 5 µg/mL), TLR 1/2 (Pam3CSK4, 5 µg/mL) and TLR4 (LPS, 5 µg/mL), as indicated. Percentage (%) tumor killing in the treatment conditions was calculated compared to untreated conditions, where no cetuximab was added (no mab) (set at 0%). Bars represent mean ± SD; * *p* < 0.05; ** *p* < 0.01; *** *p* < 0.001, **** *p* < 0.0001, ns = not significant. (**C**) Representative images of NK cells co-cultured with VU-SCC-096 cells with or without cetuximab and/or Pam3CSK4 are shown. Pictures were taken from the culture wells with a Zeiss microscope fitted with camera using 10× magnification. The inserted magnifications in the top corners are digitally enlarged fields of the original 10× magnification.

Next, we investigated whether the cytotoxic activity of immune cells could be enhanced to improve cetuximab-mediated tumor cell killing. We previously demonstrated that cross-talk between FcγRs and TLRs on DCs profoundly increased cellular activity [27,30]. As TLR1/2, TLR2/6, TLR4 and TLR5 are the most prominently expressed surface TLRs on NK cells [28], we investigated whether the co-activation of FcγRs with these TLRs would enhance the cytotoxic activity of NK cells, resulting in improved tumor cell killing. The addition of the TLR2 ligands Pam2CSK4 or Pam3CSK4 to ADCC experiments with either PBMCs or NK cells in the presence of cetuximab induced a significant increase in the tumor cell killing of VU-SCC-096 cells (Figure 2A). When cetuximab was combined with the TLR4 agonist LPS, we observed enhanced tumor cell killing when PBMCs were used as effector cells, but not when purified NK cells were used. Monocytes, which are present in the PBMC fraction and are sensitive to TLR4 activation [34], are potentially responsible for the observed improved tumor cell killing. Importantly, when the less sensitive UM-SCC-47 cells were incubated with cetuximab in combination with TLR agonists, tumor cell killing by either PBMCs or NK cells was significantly enhanced as well (Figure 2B). Flagellin, a TLR5 ligand, enhanced neither the tumor cell killing of VU-SCC-096 cells nor of UM-SCC-47 cells (data not shown). Microscopic images of an ADCC experiment in the presence of cetuximab showed NK cell clustering with target cells, which was enhanced in the presence of TLR agonists (Figure 2C).

### 2.3. TLR2 Agonists Enhance the Number of Cytotoxic NK Cells in Combination with Cetuximab

**Figure 3:**
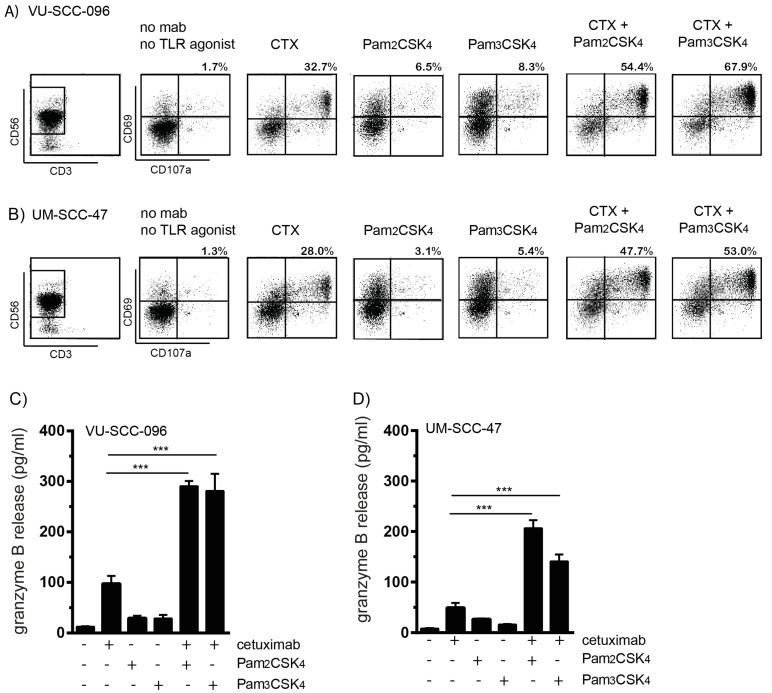
TLR2 agonists enhance the number of cytotoxic NK cells in combination with cetuximab. NK cells were harvested from ADCC experiments with the HNSCC cell lines (**A**) VU-SCC-096 or (**B**) UM-SCC-47 after 4 h incubation. NK cells (gated as CD3-CD56+ cells) were analyzed for percentages (%) of CD69 (activation marker) and CD107a (degranulation marker) double positive NK cells (upper right square). ADCC conditions included no stimulation (no mAb, no TLR agonist), cetuximab (CTX), Pam2CSK4 or Pam3CSK4. Combined treatments of cetuximab with TLR2 agonists are indicated by CTX + Pam2CSK4 or CTX + Pam3CSK4. Granzyme B release was measured in supernatants of ADCC experiments with NK cells and (**C**) VU-SCC-096 cells or (**D**) or UM-SCC-47 cells. Bars represent mean ± SD; *n* > 3; *** *p* < 0.001.

As the improvement of NK-cell-mediated ADCC was most pronounced by TLR2 agonists, we investigated whether this was due to increased cytotoxic capacity. Therefore, the percentages of CD69+CD107a+ NK cells and the release of granzyme B were assessed in the ADCC experiments. The incubation of VU-SCC-096 cells or UM-SCC-47 cells with NK cells resulted in less than 2% levels of CD69+CD107a+ NK cells (Figure 3A,B). The addition of the TLR2 ligands Pam2CSK4 or Pam3CSK4 to the co-cultures induced NK cell activation, represented by the number of CD69+ NK cells, but limited NK cell cytotoxicity, represented by the number of CD69+CD107a+ NK cells (less than 10%). The addition of cetuximab to the co-cultures induced around 30% levels of CD69+CD107a+ NK cells. The combination of cetuximab with the TLR2 agonists enhanced the percentages of CD69+CD107a+ NK cells even further, up to almost 68% and 53% for VU-SCC-096 cells and UM-SCC-47 cells, respectively. When PBMCs were used as effector cells in the ADCC experiments with either VU-SCC-096 cells or UM-SCC-47 cells, in the presence of both cetuximab and TLR2 agonists, enhanced percentages of CD69+CD107a+ NK cells were observed as well (Figure S2). Additionally, modest granzyme B release was induced, when either cetuximab or TLR2 agonists alone were added to the co-cultures of either VU-SCC-096 cells or UM-SCC-47 cells with NK cells. The combination of cetuximab with TLR2 agonists synergistically induced the release of granzyme B (Figure 3C,D). Thus, when both FcγRs and TLR2 were triggered, more functional cytotoxic NK cells were induced.

**Figure 4:**
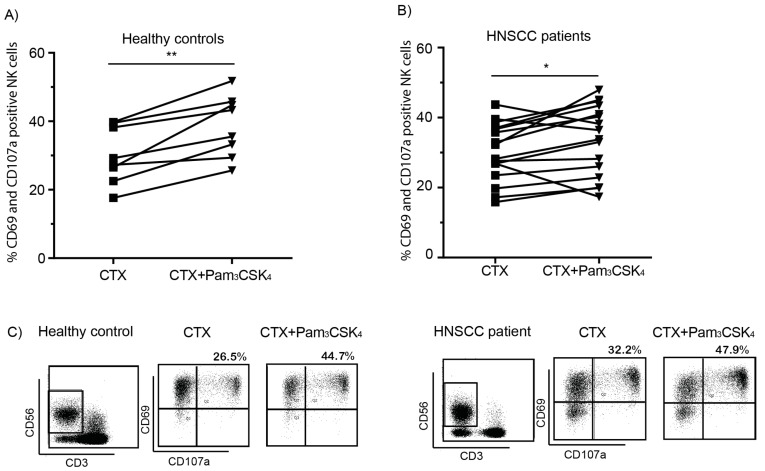
HNSCC patient PBMCs are able to induce tumor cell killing. PBMCs isolated from (**A**) 8 healthy controls and (**B**) 16 HNSCC patients were used in ADCC experiments with VU-SCC-096 cells. PBMCs were harvested after 4 h incubation and analyzed for percentages (%) of CD69 (activation marker) and CD107a (degranulation marker) double positive NK cells (gated as CD3-CD56+ cells). Graphs depict % CD69+CD107a+ NK cells in the presence of cetuximab (CTX, 0.5 µg/mL) or cetuximab in combination with Pam3CSK4 (5 µg/mL). * *p* < 0.05; ** *p* < 0.01. (**C**) Representative flow cytometry dot plots of NK cells stained for CD69 and CD107a of a healthy control in (**A**) and an HNSCC patient in (**B**).

Next, we determined whether the cytotoxic capacity of NK cells isolated from HNSCC patients was enhanced with TLR2 stimulation. PBMCs of 16 HNSCC patients, who were diagnosed with stage II to IV primary tumors in the oral cavity, oropharynx or larynx, were used to analyze the number of cytotoxic NK cells in the presence of different stimuli. We observed that PBMCs from both healthy donors (Figure 4A,C) and HNSCC patients (Figure 4B,C), that were co-cultured with VU-SCC-096 cells in the presence of cetuximab and Pam3CSK4, significantly increased the percentage of CD69+CD107a+ NK cells.

### 2.4. Combination of Cetuximab with TLR2 Agonists Induces Profound Pro-Inflammatory Responses by PBMCs

**Figure 5:**
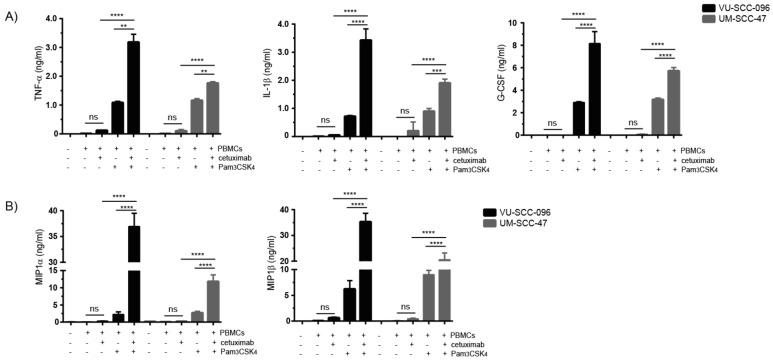
Combination of cetuximab with TLR2 agonists induces profound pro-inflammatory responses by PBMCs. ADCC experiments with the HNSCC cell lines VU-SCC-096 (black bars) and UM-SCC-47 (grey bars) were performed with PBMCs in the absence or presence of cetuximab (0.5 µg/mL) and/or Pam3CSK4 (5 µg/mL). After 24 h, supernatants were harvested and used for (**A**) cytokine and (**B**) chemokine analysis. Bars represent mean ± SD; *n* = 2; ** *p* < 0.01; *** *p* < 0.001; **** *p* < 0.0001; ns = not significant.

As a synergistic improvement of NK cell cytotoxic activity was observed when cetuximab treatment was combined with pro-inflammatory TLR2 agonists, we investigated whether FcγR-TLR2 cross-talk enhanced the release of pro-inflammatory mediators as well. Therefore, levels of various immunomodulating factors were analyzed in the supernatants of ADCC experiments. Minimal release of pro-inflammatory cytokines was detected in the majority of supernatants in the presence of cetuximab (Figure 5A and Figure S3A). However, the simultaneous stimulation of PBMCs with cetuximab and Pam3CSK4 resulted in the synergistic release of the pro-inflammatory cytokines TNF-α, IL-1β and G-CSF (Figure 5A). In line with our cytotoxicity data, the release of pro-inflammatory cytokines after stimulation with cetuximab and Pam3CSK4 was lower in ADCC experiments with UM-SCC-47 cells as target cells compared to VU-SCC-096 cells. This suggests a higher threshold for the activation of immune cells via FcγR-TLR2 cross-talk when incubated with UM-SCC-47 cells. GM-CSF, which recruits and activates myeloid cells and promotes angiogenesis, and the immunoregulating cytokines IL-6 and IL-10, were upregulated after Pam3CSK4 stimulation with no or minimal effect caused by the addition of cetuximab (Figure S3A). There were no differences in the levels of immunosuppressive TGF-β after stimulation (data not shown). Additionally, the release of IL-8 was already highly induced after cetuximab stimulation and did not change following the addition of Pam3CSK4 (data not shown). No release of the T-cell (polarizing) cytokines IFN-γ, IL-12, IL-2 and IL-23 was observed in any of the conditions (data not shown).

The most pronounced levels of chemokines that were secreted in the supernatants in the presence of cetuximab and Pam3CSK4 were the granulocyte chemoattractants MIP1α and MIP1β (Figure 5B). As observed with pro-inflammatory cytokines, reduced release of these chemokines was observed when UM-SCC-47 cells were used as target cells compared to VU-SCC-096 cells. Low levels of the B-cell chemoattractant CXCL13 and the T-cell chemoattractant RANTES were observed after cetuximab stimulation, although the release increased slightly following the addition of Pam3CSK4 (Figure S3B). High levels of the monocyte chemoattractant MCP-1 were already observed after cetuximab stimulation, which did not change after the addition of Pam3CSK4 (data not shown). No release of the eosinophil chemoattractant Eotaxin was detected in any of the conditions (data not shown). Together, these data indicate that the addition of TLR2 agonists to cetuximab treatment induces the synergistic release of the pro-inflammatory cytokines TNF-α, IL-1β and G-CSF, as well as the chemokines MIP1α and MIP1β.

### 2.5. Treatment of Tumor-Bearing Mice with Cetuximab and Pam3CSK4 Results in Prolonged Survival

**Figure 6:**
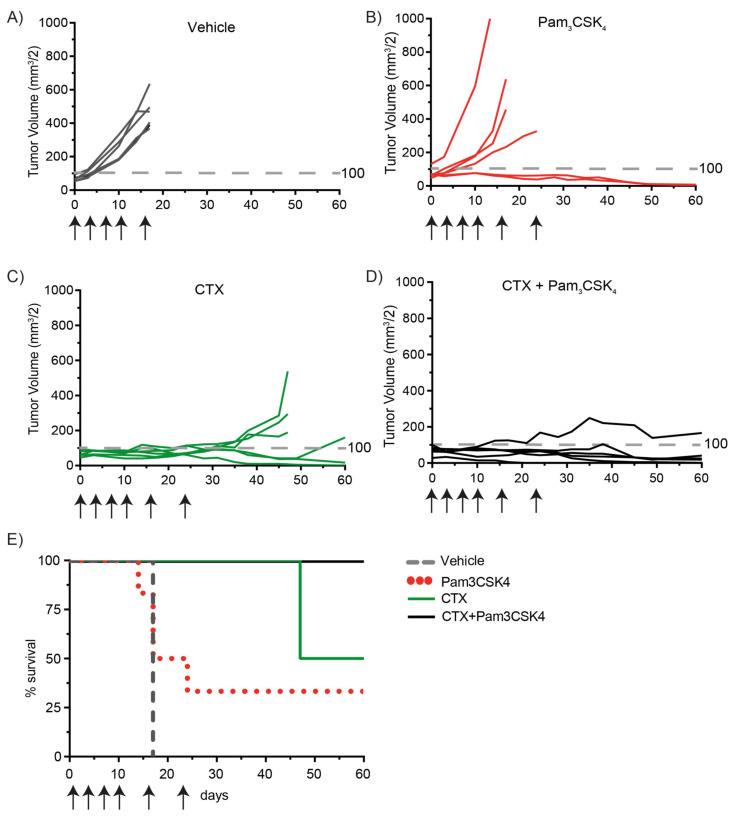
Treatment of tumor-bearing mice with cetuximab and Pam3CSK4 results in prolonged survival. Nude mice (six/group) were subcutaneously injected with ADCC-insensitive UM-SCC-47 cells in both flanks. When tumors reached a tumor volume of approximately 70 mm^3^, intraperitoneal treatments were started (day 0). Mice were treated with (**A**) PBS (vehicle), (**B**) Pam3CSK4 (50 µg/mouse), (**C**) cetuximab (CTX, 1 mg/mouse) or (**D**) cetuximab with Pam3CSK4. Treatment days are indicated with arrows (day 0, 3, 7, 10, 17 and 24). Tumor volume is depicted as the mean of two tumors (both flanks, mm^3^) per mouse. Fragmented grey line represents a tumor volume of 100 mm^3^. (**E**) Relative survival rate of mice in the different treatment groups (six mice/group set at 100%).

Finally, we explored whether TLR1/2 co-activation could improve cetuximab-mediated tumor elimination in tumor-bearing mice, as well as the infiltration of immune cells in the tumors. Of note, IgG1 antibodies, such as cetuximab, and Pam3CSK4 bind FcγRs and TLR2, respectively, on murine immune cells [35,36]. Mice were subcutaneously injected with UM-SCC-47 cells, which was used as a model to determine the efficacy of combination treatment in vivo, as this cell line showed low sensitivity to NK-cell-mediated ADCC, when treated with cetuximab in vitro. When the tumor volume reached approximately 70 mm^3^, intraperitoneal treatment with cetuximab and/or Pam3CSK4 was started and continued at indicated intervals. All tumor-bearing mice that were treated with PBS were sacrificed on day 18 due to rapid tumor outgrowth (Figure 6A). Mice that received Pam3CSK4 displayed more diversity in tumor outgrowth. Four mice experienced rapid tumor outgrowth and were sacrificed between day 14 and 24, while the two remaining mice demonstrated tumor regression until the end of the experiment (day 60) (Figure 6B). All mice treated with cetuximab with or without treatment with Pam3CSK4 displayed restrained tumor growth (Figure 6C,D). However, tumor volume rapidly increased in 50% of the mice treated with cetuximab after approximately 30 days. These mice were sacrificed at day 47 due to large tumor volume or tumor ulceration (Figure 6C). The remaining mice displayed complete tumor clearance or minimal tumor outgrowth until the end of the experiment. Importantly, five out of six mice treated with both cetuximab and Pam3CSK4 showed complete tumor clearance or tumor regression to less than the start volume of the tumors (Figure 6D). Thus, all tumor-bearing mice treated with a combination of cetuximab and Pam3CSK4 survived up to 60 days after the start of the treatment, in contrast to untreated mice and mice treated with either cetuximab or Pam3CSK4 alone (Figure 6E).

**Figure 7:**
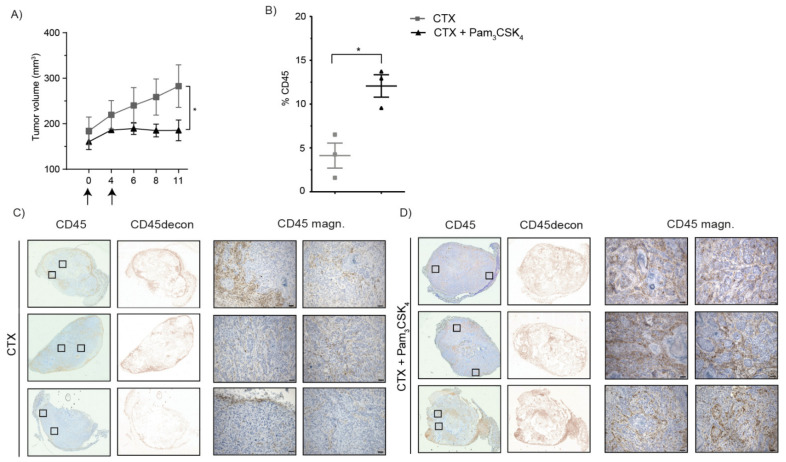
Treatment with cetuximab and Pam3CSK4 facilitates infiltration of immune cells into tumors. Nude mice (three/group) were subcutaneously injected with ADCC-insensitive UM-SCC-47 cells in both flanks. Tumors were harvested at day 11 after treatment (cetuximab or cetuximab with Pam3CSK4 at day 0 and 4), *n* = 1. (**A**) Tumor volume is depicted as the mean of two tumors (both flanks, mm^3^) ± SEM; * *p* < 0.05 is considered significant. (**B**–**D**) Tumors (one/mouse) were stained for the presence of CD45 (general immune cell marker, brown staining) and counterstained with hematoxylin. (**B**) Percentage (%) CD45 staining within the tumor area; * *p* < 0.05 is considered significant. (**C**,**D**) Deconvolution of CD45-DAB staining from hematoxylin staining of the tumors described in (**B**) is shown in the second column (CD45decon). Two magnifications per tumor (of CD45-DAB and hematoxylin staining), indicated by squares in the first column, are shown in the third and fourth column (CD45 magn.) for CTX-treated (**C**) and CTX + Pam3CSK4-treated animals (**D**). Scale bars represent 50 µm.

To investigate immune cell infiltration in the tumors, we performed a similar in vivo experiment and sacrificed all tumor-bearing mice at day 11 before tumor regression was observed (Figure 7A). The presence of immune cells was demonstrated by CD45 (immune cell marker) staining (Figure 7B–D). Quantification of the percentages of CD45+ immune cells relative to the tumor area demonstrated significantly increased immune cell presence in the tumors of mice treated with cetuximab and Pam3CSK4 compared to cetuximab alone (Figure 7B). Moreover, in cetuximab-treated mice, immune cells were mostly found at the border of the tumors (Figure 7C) compared to the massive influx of CD45+ cells into the tumor mass of mice treated with cetuximab and Pam3CSK4 (Figure 7D). Of note, staining and quantification of the tumor cell marker CD44v6 demonstrated no significant differences, in terms of the percentages of tumor cells, between the treatment groups (Figure S4A,B). In conclusion, treatment of tumor-bearing mice with cetuximab and Pam3CSK4 resulted in improved tumor cell clearance, which was associated with enhanced immune cell infiltration into the tumor mass, supporting the beneficial immunostimulatory activity of Pam3CSK4 during anti-cancer immunotherapy.

## 3. Discussion

Many solid malignancies harbor an immunosuppressive TME, favoring tumor survival due to the evasion of anti-tumor immune responses. Therefore, reinforcing immune responses could be a potential strategy to improve cancer treatment. Currently, several approaches are investigated to improve immunotherapy in HNSCC, focusing on enhanced immune recognition, modulation of the immunosuppressive TME or regulation of immune checkpoint molecules [37]. Strategies to stimulate anti-tumor immune responses include treatment with cytokines such as IL-2, IL-15 and IFN-α, which have been shown to augment the efficacy of immune cells in vitro and in vivo [21,29,38]. However, this treatment strategy is associated with severe toxicity in patients, i.e., a first-in-human clinical trial in which patients with metastatic cancer were treated with recombinant IL-15 showed significant toxicity at the effective treatment dose [39]. Yet, treatment did induce activation of CTLs and NK cells. Therefore, linking IL-15 treatment to a tumor-targeting agent could reduce off-target toxicity. Currently, the IL-15 fusion protein ALT-803, in combination with rituximab, is being tested in a clinical trial in patients with indolent B cell non-Hodgkin lymphoma (NCT02384954). ALT-803 was shown to induce NK cell activation in a phase I study in patients with solid cancers, which included patients with HNSCC (NCT01946789) [40]. As with IL-15, severe toxicity is a problem for treatment with high doses of IL-2. Various clinical studies are ongoing using a PEGylated IL-2 agonist binding CD122 (bempegaldesleukin, NKTR-214) combined with anti-PD-L1 or anti-PD-1 in solid metastatic cancers (NCT03138889), one of which is testing a combined treatment with intratumoral injection of a TLR agonist (NKTR-262) (NCT03435640).

TLRs, which possess a natural immunostimulatory capacity, are explored as a potential target to enhance anti-tumor immune responses [26,27,28,29]. The main TLR agonists that have been investigated in this setting target intracellular TLR7/8 or TLR9 to stimulate antigen uptake and presentation by DCs, thereby boosting CTL priming [28]. However, the use of TLR agonists in combination with antibody therapy is limited. The combination of cetuximab with either TLR3 or TLR8 stimulation induced increased NK-cell-mediated ADCC of head and neck cancer cells compared to cetuximab alone [41,42]. In a phase I study, HNSCC patients were treated with cetuximab in combination with motolimod, a small molecule TLR8 agonist [43]. Treatment resulted in increased infiltration of pro-inflammatory M1 macrophages and CTLs, whereas infiltration of regulatory T-lymphocytes and MDSCs was decreased. Additionally, the expression of immunosuppressive markers was reduced. However, treatment had no effect on survival [44,45]. Interestingly, clinical benefit was observed in HPV+ patients, as well as patients with injection site reactions. This suggests that combination treatment could be beneficial in a subgroup of patients. Toxicities of the TLR agonist treatment due to widespread immune cell activation can be overcome by local, intra- or peritumoral injections [46,47,48], or by conjugation of TLR agonists to antibodies [49].

Here, we demonstrate that antibody-mediated immunotherapy combined with TLR2 stimulation improves the cytotoxic function of NK cells and subsequently enhances the elimination of tumor cells in vitro. Importantly, the improved cytotoxicity is not only observed with NK cells isolated from healthy donors, but with those isolated from HNSCC patients as well. Moreover, we demonstrate enhanced tumor clearance in vivo after combination treatment, associated with significantly increased immune cell infiltration into the tumors. Thus, combination of antibody-mediated immunotherapy with TLR2 targeting could represent an opportunity to improve NK cell cytotoxicity, which is frequently impaired in HNSCC patients, resulting in enhanced anti-tumor immune responses. As such, this treatment strategy may increase the success rate of cetuximab treatment, thereby enhancing the survival of HNSCC patients. Moreover, therapeutic efficacy could potentially be further enhanced in combination with immune checkpoint inhibitors.

The augmented cytotoxic capacity of the NK cells coincided with robust immune responses indicated by the release of pro-inflammatory cytokines and chemokines. After activation, NK cells secrete pro-inflammatory cytokines, including IFN-γ, TNF-α and IL-1β, as well as chemokines, such as MIP1α, MIP1β and RANTES [50]. The combination of cetuximab with Pam3CSK4 induced the synergistic release of several pro-inflammatory cytokines in our ADCC experiments. This is in line with recent findings that cross-talk between antibody-binding FcγRs and pathogen-recognizing TLRs on immune cells results in pronounced pro-inflammatory cytokine responses [22,51,52,53]. In contrast, limited changes in anti-inflammatory cytokines were detected. Substantial release of the granulocyte-attracting chemokines MIP1α and MIP1β was observed after stimulation with both cetuximab and Pam3CSK4, while the release of RANTES and CXCL13, chemokines that attract T-lymphocytes and B-lymphocytes, respectively, was less pronounced. Interestingly, neutrophils are potent effector cells in anti-tumor immune responses [54,55]. Therefore, neutrophils may contribute to the elimination of tumor cells after antibody-mediated immunotherapy. The combination of cetuximab treatment with Pam3CSK4-mediated TLR2 stimulation enhanced cytotoxicity, as well as cytokine production by NK cells, against both the ADCC-sensitive cell line VU-SCC-096 and the ADCC-insensitive cell line UM-SCC-47, although tumor cell killing and cytokine production remained lower in the latter. Neither EGFR nor PD-L1 or PD-L2 expression levels could explain the difference in sensitivity to cetuximab-mediated ADCC between the cell lines. Future research should focus on identifying the intrinsic factors of tumor cells that are responsible, as this might lead to discovery of novel immunosuppressive targets or mechanisms of resistance.

Enhanced tumor cell killing by immune cells after treatment with cetuximab in combination with TLR2 stimulation in vitro is supported by the in vivo data. Treatment of mice bearing ADCC-insensitive UM-SCC-47 tumors with both cetuximab and Pam3CSK4 resulted in significantly decreased tumor growth or even complete tumor regression. Importantly, tumors of mice treated with a combination of cetuximab and Pam3CSK4 showed increased immune cell infiltration compared to those of cetuximab-treated mice. This suggests that tumor clearance in vivo is associated with infiltration of immune cells and that combination therapy, in mice, could convert a cold, immune-excluded TME into a hot, immune-infiltrated TME. Therefore, such a treatment could also potentially enhance the clinical effect of subsequent treatment with immune checkpoint inhibitors.

In summary, combination treatment, consisting of antibody-mediated immunotherapy combined with TLR2 stimulation, represents a promising immunomodulatory approach to overcome the immunosuppressive TME, resulting in improved immune cell infiltration within the tumor and the subsequent elimination of tumor cells. This may enhance clinical responses to antibody-mediated immunotherapy, thereby augmenting the survival of HNSCC patients.

## 4. Materials and Methods

### 4.1. Antibodies and Reagents

Cetuximab (Erbitux) was purchased from Merck KGaA (Darmstadt, Hessen, Germany). Recombinant IL-15 was obtained from PeproTech (Rocky Hill, NJ, USA, cat. no. 200-15). The TLR ligands Pam2CSK4, Pam3CSK4 and flagellin were purchased from Invivogen (San Diego, CA, USA, cat. no. tlrl-pm2s-1, tlrl-pms and tlrl-bsfla) and lipopolysaccharide (LPS) from Sigma-Aldrich (Saint Louis, MO, USA, cat. no. L4391). Monensin (GolgiStop) was purchased from BD Biosciences (San Diego, CA, USA, cat. no. 554724). The monoclonal antibodies anti-CD69-BV412 (clone FN50, cat. no. 310930) and anti-CD107a-APC (clone H4A3, cat. no. 328620) were purchased from BioLegend (San Diego, CA, USA), while anti-CD56-PE (clone MY31, cat. no. 345810), anti-CD3-PerCP-Cy5.5 (clone SK7, cat. no. 332771), anti-PD-L1-BV786 (clone MIH1, cat. no. 563739), mIgG1-BV786 (clone X40, cat. no. 563330), anti-PD-L2-BV711 (clone MIH18, cat. no. 564258) and mIgG1-BV711 (clone X40, cat. no. 563044) were obtained from BD Biosciences. Goat polyclonal IgG F(ab)_2_ anti-hIgG-PE was purchased from Bio-Connect (Huissen, The Netherlands, cat. no. A59star97PE).

### 4.2. Cell Culture

#### 4.2.1. HNSCC Cell Lines

HNSCC cell lines VU-SCC-040, VU-SCC-096, VU-SCC-120 and VU-SCC-147 were established at Amsterdam UMC, location VUmc (Amsterdam, Noord-Holland, The Netherlands) [56,57]. Cell lines UM-SCC-11B, UM-SCC-14C, UM-SCC-22A, UM-SCC-38 and UM-SCC-47 were a kind gift from Prof. Dr. T.E. Carey (University of Michigan, Ann Arbor, MI, USA) [58]. The FaDu cell line was purchased from ATCC (Manassas, VI, USA). Cell lines were authenticated by microsatellite PCR profiling and TP53 sequencing. Cell lines VU-SCC-147 and UM-SCC-47 were HPV+, while all other cell lines were HPV-, as confirmed by a GP5+/6+ DNA PCR [59]. The cell lines were cultured in Dulbecco’s Modified Eagle’s Medium (DMEM) supplemented with 5% fetal bovine serum (FBS) (Gibco, cat. no. 10270-106) and 2 mM L- glutamine (Lonza, cat. no. BE-17-605E), and were free from mycoplasma contamination. Cultures were maintained at 37 °C in a humidified atmosphere with 5% CO_2_.

#### 4.2.2. Immune Cells

Human PBMCs were isolated from heparinized blood of healthy donors or buffycoats by means of a standard Ficoll density centrifugation protocol (LymphoprepTM, Axis Shield, Oslo, Norway, cat. no. 1114544). The PBMCs of HNSCC patients and healthy donors were isolated from heparinized blood using Leucosep tubes (Greiner Bio-One, Kremsmünster, Austria, cat. no. 227290). Human NK cells were isolated from PBMCs using an NK cell isolation kit (Miltenyi Biotec, Bergisch Gladbach, Germany, cat. no. 130-092-657) according to the manufacturer’s protocol. The purity of the NK cells was at least 95%. PBMCs and NK cells were cultured in RPMI 1640 supplemented with 10% FBS and 2 mM L-glutamine. Cultures were maintained at 37 °C in a humidified atmosphere with 5% CO_2_. Tubes with freshly drawn heparinized blood of healthy donors or buffycoats were purchased from Sanquin (Amsterdam, The Netherlands). The Institutional Review Board of Amsterdam UMC, location VUmc, approved the collection of blood and the use of immune cells from HNSCC patients, who provided informed consent (2008.071|A2016.035). Clinical details are provided in Supplementary Table S1. Pathological TNM staging was used for patients treated with surgery, while clinical TNM staging was used for patients treated otherwise.

### 4.3. ADCC Assay

HNSCC tumor cells were seeded in 96-well flat-bottom plates. The next day, immune cells were added at an effector-to-target (E:T) ratio of 60:1 or 5:1 for PBMCs or NK cells, respectively. Cetuximab (0–2 µg/mL), IL-15 (10 ng/mL) and synthetic agonists for TLR 1/2 (Pam3CSK4), TLR 2/6 (Pam2CSK4), TLR4 (LPS) and TLR 5 (flagellin) (all 5 µg/mL, unless otherwise stated) were added when indicated and were diluted in the culture medium to obtain the indicated concentrations. After 4–24 h, supernatants were collected, centrifuged twice for 5′ at 300× *g* and stored at −20 °C. Plates were washed with phosphate-buffered saline (PBS) (Lonza, Basel, Switzerland, cat. no. BE17-516F) and cell viability was determined via the CellTiter-Blue Cell Viability Assay (Promega, Leiden, The Netherlands, cat. no. G8080) according to the manufacturer’s protocol. Fluorescence was measured using a GloMax^®^-Multi Detection System (Promega). Fluorescence of treated samples was compared to that of untreated samples to calculate the percentage of killing using the formula (1 − (mean fluorescence treated/mean fluorescence untreated)) × 100%.

### 4.4. Flow Cytometry

For the analysis of degranulation, tumor cells were cultured overnight in 96-well flat-bottom plates. The next day, rested immune cells were added to the tumor cells with or without cetuximab, TLR ligands and anti-CD107a antibody. Between 30 and 60′ after the start of the ADCC experiment, monensin was added to the culture. After harvesting, cells were incubated with primary antibodies in PBS with 0.5% BSA (Sigma-Aldrich, Saint Louis, MO, USA) for 45′ on ice and, when indicated, incubated with a secondary antibody for 60′. After staining, cells were measured on an LSRFortessa™ cell analyzer (BD Biosciences) and analyzed with DIVA software version 8.0 (BD Biosciences, San Diego, CA, USA).

### 4.5. Multiplex Protein Analysis

Supernatants of ADCC experiments (stored at −20 °C, see above) were used for protein analysis. A custom-made multiplex assay for CXCL13, Eotaxin/CCL11, G-CSF, GM-CSF, IFN-γ, IL-1β, IL-10, IL-12p70, IL-2, IL-23, IL-6, IL-8, IP10/CXCL10, MCP-1/CCL2, MIP1α/CCL3, MIP1β/CCL4, RANTES/CCL5 and TNF-α from R&D Systems (Minneapolis, MN, USA) was performed using LUMINEX technology (LX200™, Millipore, Burlington, MA) according to the manufacturer’s protocol. The cytokines IFN-γ, TGF-β and TNF-α (eBioscience, San Diego, CA, USA, cat. no. 88-7316-22, 88-8350-88, 39-8329, respectively) and granzyme B (MABTECH, Stockholm, Sweden, cat. no. 3485-3-250/6-250) were separately analyzed in sandwich ELISAs according to the manufacturer’s protocol.

### 4.6. In Vivo Experiments

Nude mice (athymic nu/nu, female, 6–8 weeks old) were obtained from Envigo (Horst, Limburg, The Netherlands) and were kept in filter top cages under sterile conditions in standardized environmental conditions. UM-SCC-47 tumor cells (2 × 10^6^ cells) were subcutaneously injected in both flanks. When tumors reached an average size of 70 mm^3^ or 150 mm^3^ (as indicated in the graphs), intraperitoneal treatment was started. Cetuximab (1 mg/mouse) was injected with or without Pam3CSK4 (50 µg/mouse). PBS was used as a vehicle control. Mice were sacrificed when the tumor reached 5x the start volume in one of the flanks and/or displayed ulceration, or when mice displayed body weight loss ≥ 20% or moribund appearance. Tumor volume was measured with electronic calipers (V = (L × W × H) × 0.5, where V = volume, L = length, W = width, H = height) and calculated as the mean of both tumors per mouse. For analysis of immune cell infiltration into the tumors, tumors were harvested at day 11 and embedded in paraffin. All animal experiments were performed according to the NIH Principles of Laboratory Animal Care and Dutch national law (Wet op de Dierproeven, Stb 1985, 336).

### 4.7. Immunohistochemistry

Paraffin-embedded tumor blocks were cut to sections of 3 µm thickness, deparaffinized and rehydrated by incubation in xylene and graded concentrations of ethanol. Antigen retrieval was performed by heat-induced epitope retrieval in Tris-EDTA (pH 9) buffer. Endogenous peroxidases were blocked by immersion in 3% hydrogen peroxide in PBS for 10′, after which samples were blocked with 10% normal goat serum (NGS) (DAKO, Carpinteria, CA, cat. no. X0902) for 30′ at RT. Next, slides were incubated for 1 h at RT with rabbit anti-human/mouse CD45 (Abcam, Cambridge, UK, cat. no. Ab10558) (1:5000 in PBS with 1% BSA and 2% NGS) or rabbit immunoglobulin fraction (DAKO, cat. no. X0903) as an isotype control, after which slides were washed twice with PBS and incubated with Poly-HRP-anti-mouse/rabbit IgG (BrightVision IHC Detection Kit, Immunologic, Duiven, The Netherlands, cat. no. DPVB55-HRP) for 30′. Staining was visualized with 3,3’-Diaminobenzidine (DAB) (Sigma, cat. no. D5637). For the anti-CD44v6 staining, slides were blocked with 10% normal rabbit serum (DAKO) and incubated with the chimeric anti-human CD44v6 antibody (10 µg/mL) (own production [60]), followed by staining with rabbit-anti-human IgG-HRP (DAKO). All tissue sections were briefly rinsed in water and counterstained with hematoxylin (Merck) for 1′, after which slides were gently rinsed with distilled water and mounted in Kaiser Glycerol gelatin (Merck). Tile scans were made using a LEICA 6000 DM microscope (LEICA, SOLMs, Germany) (5x scanning objective) and Leica software. Quantification of CD45 and CD44v6 staining was determined using Fiji software. The region of interest (tumor area) was manually selected, followed by the selection of color threshold settings. Measurements of staining were calculated and depicted as % of DAB staining compared to tumor area. Deconvolution of the CD45-DAB signal from the hematoxylin counterstaining was performed with the H-DAB vector mask [61].

### 4.8. Statistical Analyses

Data analysis was performed using GraphPad Prism version 6.07 (GraphPad Software, San Diego, CA, USA). Data are depicted as mean ± SD or mean ± SEM as indicated. Statistical differences were determined using two-tailed Student’s *t*-tests (2 groups) or one-way ANOVA with multiple comparisons tests (>2 groups). *p*-values < 0.05 were considered significant (* *p* < 0.05; ** *p* < 0.01; *** *p* < 0.001; **** *p* < 0.0001; ns = not significant).

## Data Availability

The data presented in this study are available in the manuscript.

## Supplementary Materials

The following are available online at https://www.mdpi.com/article/10.3390/ijms222011057/s1.
