# Supplementary material for: NK Cell-Dependent Antibody-Mediated Immunotherapy Is Improved In Vitro and In Vivo When Combined with Agonists for Toll-like Receptor 2 in Head and Neck Cancer Models
Source: Int J Mol Sci. 2021 Oct 14;22(20):11057. doi: 10.3390/ijms222011057 (PMC8541276; doi:10.3390/ijms222011057)
Supplement: Supplementary file 1 [file ijms-22-11057-s001.zip › ijms-1410136-supplementary.pdf]

NK cell-dependent antibody-mediated immunotherapy is improved *in vitro* and *in vivo* when combined with agonists for Toll-like receptor 2 in head and neck cancer models

Supplementary figure S1

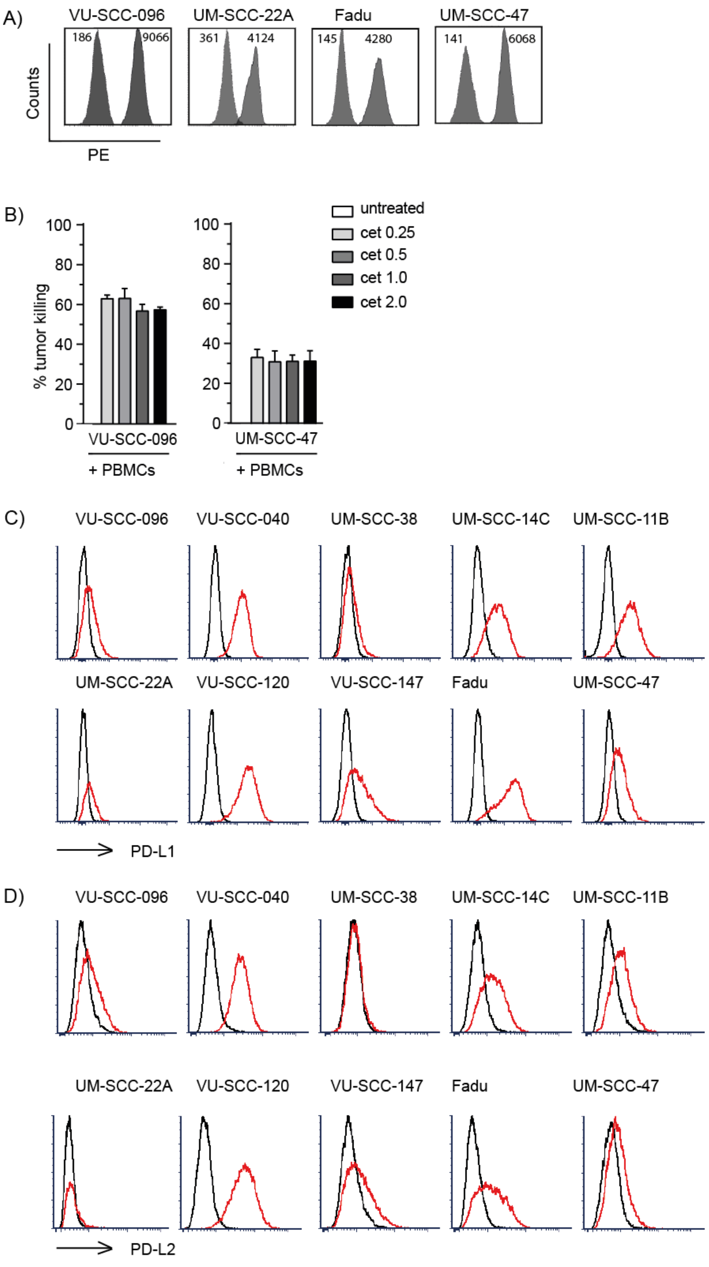

Cetuximab-mediated killing of HNSCC cells by immune cells. (**A**) EGFR expression on 4 HNSCC cell lines analyzed by flow cytometry. First peak represents staining with secondary PE-labeled antibody only. Second peak represents staining with cetuximab (0.25 µg/ml) and secondary PE-labeled antibody. Graph indicates median fluorescence intensity. (**B**) ADCC experiments with the HNSCC cell lines VU-SCC-096 and UM-SCC-47 were performed with PBMCs (E:T ratio 60:1, 24h incubation) in the presence of different concentrations of cetuximab (0.0-2.0 µg/ml). Bars indicate mean ± SD, n≥3. (**C-D**) PD-L1 (**C**) and PD-L2 (**D**) expression on HNSCC cell lines analyzed by flow cytometry. Black line represents isotype control staining, red line represents PD-L1 (**C**) or PD-L2 (**D**) staining. Graph indicates median fluorescence intensity.

Supplementary figure S2

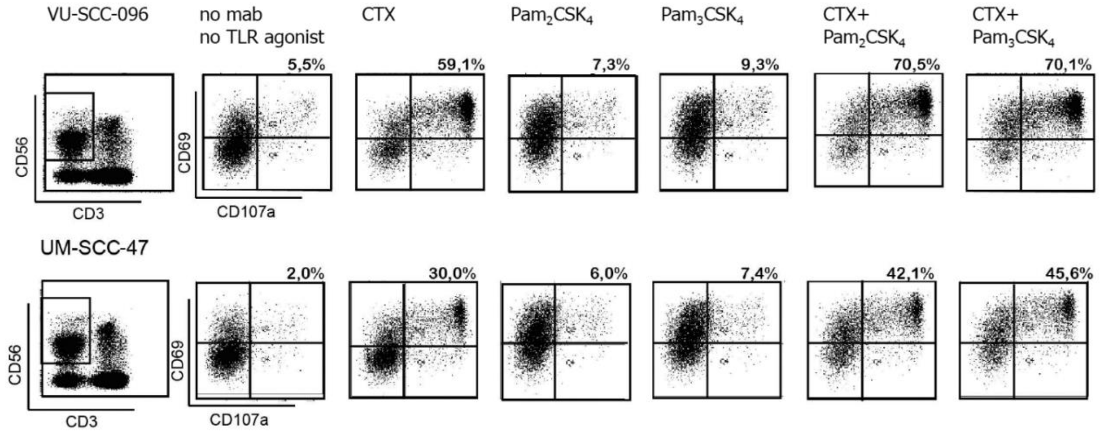

TLR2 agonists enhance NK cell cytotoxicity upon cetuximab stimulation. PBMCs were harvested from ADCC experiments with the HNSCC cell lines VU-SCC-096 (upper panels) and UM-SCC-47 (lower panels) after 24h incubation and analyzed for percentage (%) CD69 (activation marker) and CD107a (degranulation marker) double positive NK cells (gated as CD3-CD56+ cells) (upper right square). ADCC conditions included no stimulation (no mAb, no TLR agonist), cetuximab (CTX), Pam2CSK4 or Pam3CSK4. Combined treatments of cetuximab with TLR2 agonists are indicated by CTX + Pam2CSK4 or CTX + Pam3CSK4. N>3.

Supplementary figure S3

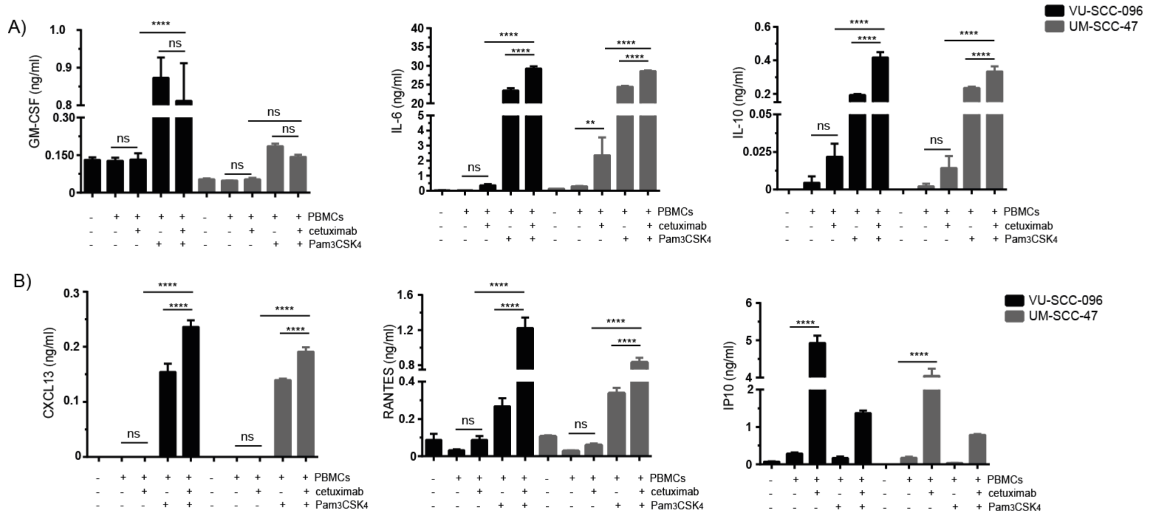

Secretory profile of PBMCs stimulated with cetuximab and a TLR2 agonist. ADCC experiments with the HNSCC cell lines VU-SCC-096 (black bars) and UM-SCC-47 (grey bars) were performed with PBMCs in the absence or presence of cetuximab (0.5 µg/ml) and/or Pam3CSK4 (5 µg/ml). After 24h supernatants were harvested and used for (**A**) cytokine and (**B**) chemokine analysis. Bars represent mean ± SD, n=2, *p<0.05 is considered significant.

Supplementary figure S4

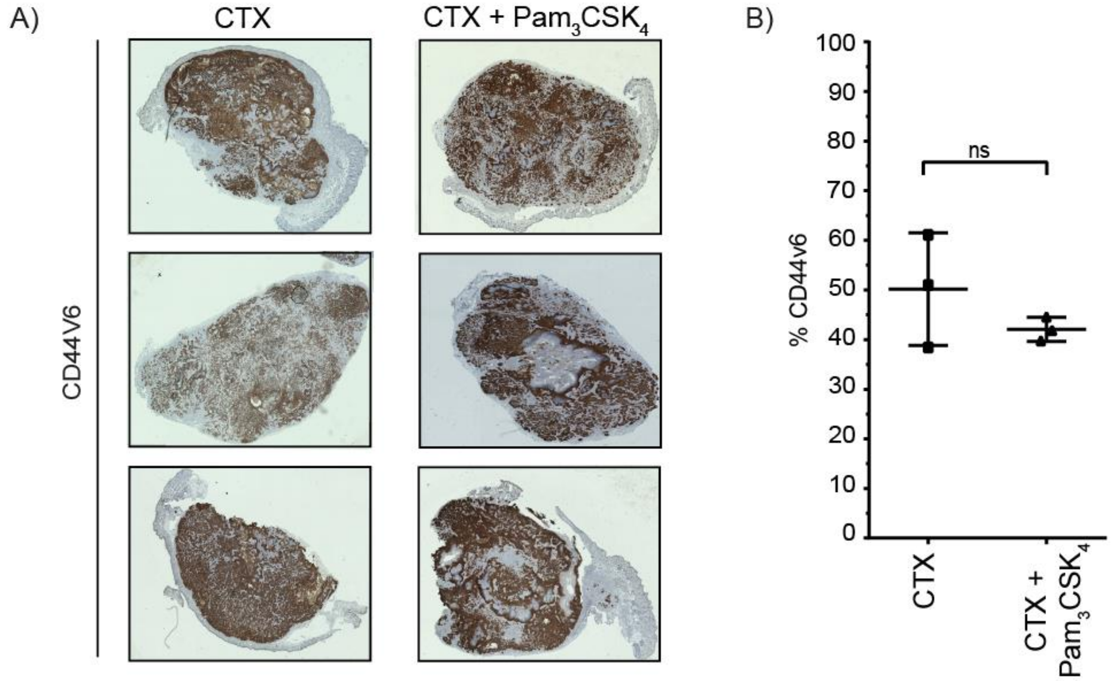

Treatment of tumor-bearing mice with cetuximab and a TLR2 agonist. (**A**) Nude mice (3/group) were subcutaneously injected with UM-SCC-47 cells in both flanks. Tumors were harvested at day 11 after treatment (cetuximab or cetuximab with Pam3CSK4s at day 0 and 4), n=1. Tumors were stained for the presence of CD44v6 (tumor cell marker, brown staining) and counterstained with haematoxylin. (**B**) Percentage (%) CD44v6 staining within the tumor area, *p<0.05 is considered significant.

Supplementary table S1

Patient no.	*Gender*	*Age at diagnosis*	*Subsite*	*TNM stage*
pt607	M	77	Glottic larynx	T3N0
pt608	F	74	Floor of mouth	T4N0
pt610	M	54	Floor of mouth	T2N0
pt617[A]	M	73	Base of tongue	T2N2b
pt618	M	69	Glottic larynx	T4aN0
pt626	F	66	Floor of mouth	T4aN0
pt628	F	74	Piriform sinus	T4bN0
pt633	M	73	Base of tongue	T4bN0
pt640[A]	M	54	Tonsil	T4aN1
pt664	M	54	Oropharynx	T3N0
pt665	F	63	Supraglottic larynx	T3N0
pt687	M	51	Floor of mouth	T2N0
pt688	M	53	Supraglottic larynx	T4aN2b
pt693	M	66	Oropharynx	T4bN0
pt695	M	72	Hypopharynx	T3N2b
pt698	M	58	Piriform sinus	T2N1

Patient and tumor characteristics. [A] HPV16+ tumors.
